# Apoptosis Effect of Girinimbine Isolated from *Murraya koenigii* on Lung Cancer Cells *In Vitro*

**DOI:** 10.1155/2013/689865

**Published:** 2013-03-13

**Authors:** Syam Mohan, Siddig Ibrahim Abdelwahab, Shiau-Chuen Cheah, Mohd Aspollah Sukari, Suvitha Syam, Noorasyikin Shamsuddin, Mohd Rais Mustafa

**Affiliations:** ^1^Department of Pharmacy, Faculty of Medicine, University of Malaya, 50603 Kuala Lumpur, Malaysia; ^2^Medical Research Centre, Jazan University, P.O. Box 114, 45 142 Jazan, Saudi Arabia; ^3^Centre for Natural Products and Drug Discovery (CENAR), Department of Pharmacology, Faculty of Medicine, University of Malaya, 50603 Kuala Lumpur, Malaysia; ^4^Faculty of Medicine & Health Sciences, UCSI University, No. 1, Jalan Menara Gading, UCSI Heights, Cheras, 56000 Kuala Lumpur, Malaysia; ^5^Department of Chemistry, Faculty of Science, University Putra Malaysia, 43400 Serdang, Malaysia; ^6^UPM-MAKNA Cancer Research Laboratory, Institute of Bioscience, University Putra Malaysia, 43400 Serdang, Malaysia

## Abstract

*Murraya koenigii* Spreng has been traditionally claimed as a remedy for cancer. The current study investigated the anticancer effects of girinimbine, a carbazole alkaloid isolated from *Murraya koenigii* Spreng, on A549 lung cancer cells in relation to apoptotic mechanistic pathway. Girinimbine was isolated from *Murraya koenigii* Spreng. The antiproliferative activity was assayed using MTT and the apoptosis detection was done by annexin V and lysosomal stability assays. Multiparameter cytotoxicity assays were performed to investigate the change in mitochondrial membrane potential and cytochrome c translocation. ROS, caspase, and human apoptosis proteome profiler assays were done to investigate the apoptotic mechanism of cell death. The MTT assay revealed that the girinimbine induces cell death with an IC_50_ of 19.01 **μ**M. A significant induction of early phase of apoptosis was shown by annexin V and lysosomal stability assays. After 24 h treatment with 19.01 **μ**M of girinimbine, decrease in the nuclear area and increase in mitochondrial membrane potential and plasma membrane permeability were readily visible. Moreover the translocation of cytochrome c also was observed. Girinimbine mediates its antiproliferative and apoptotic effects through up- and downregulation of apoptotic and antiapoptotic proteins. There was a significant involvement of both intrinsic and extrinsic pathways. Moreover, the upregulation of p53 as well as the cell proliferation repressor proteins, p27 and p21, and the significant role of insulin/IGF-1 signaling were also identified. Moreover the caspases 3 and 8 were found to be significantly activated. Our results taken together indicated that girinimbine may be a potential agent for anticancer drug development.

## 1. Introduction

Lung cancer is one of the leading causes of cancer related deaths worldwide, which has a high incidence of recurrence. It has been estimated that approximately 1.4 million are diagnosed every year and more than 1 million people die annually, 12% of which are new cases [1]. Nonsmall cell lung cancer (NSCLC) constitutes majority of lung cancers, which comprises more than 80% of total diagnoses [2]. Although chemotherapy and radiation therapy are available to treat NSCLC, they are largely ineffective and highly toxic with a low survival profile [3]. This toxicity and resistance to the current chemotherapy made researchers focus on new drug candidates, targeting apoptosis, a programmed cell death, as physiological process that provides an effective, noninflammatory way to remove redundant or damaged cells from tissues thereby securing tissue homeostasis [4]. A multitude of signals activated by variable triggers, such as growth factors, cell-cell interactions, changing nutrient conditions, hypoxic conditions, and cytotoxic damage, affect the status of the apoptotic machinery [5], and it is an effective means of treating cancer, including NSCLC [6]. The literature on drug discovery published to date reveals that the knowledge of ethnopharmacology and traditional medicine has contributed a lot to development of novel clinical agents.

“Ayurveda,” an Indian system of medicine, is gaining greater attention and reputation in many regions of the world due to increased acceptance to the scientific evidence base and the essence of vigorous research. Depending on the mode of action, the Ayurvedic pharmacology system classifies medicinal plants into different groups. As per Ayurveda, plants identified as “Rasayanas” have various pharmacological properties such as being immunostimulant, anticancer, tonic, neurostimulant, antiaging, antibacterial, antiviral, and antirheumatic [7]. The use of “Rasayana” as a body resistance enhancer has been described in detail in a distinct section of Ayurveda. *Murraya koenigii*, an aromatic small tree, belonging to the citrus family, Rutaceae, that grows widely in East Asia, is one such plant which is reported as tonic and used in various disease conditions [8]. The traditional medical literature describes its potential role as a remedy for cancer [9]. Moreover, other uses such as antioxidant properties and antidiabetic, antifungal, antibacterial, antidysentery, and antidiarrhea effects have also been investigated [10].

The main constituents reported from the plants are sterols, aminoacids, glycosides, proteins, and flavonoids. Apart from these, many carbazole alkaloids also have been identified. It has been reported that carbazole alkaloids present in the plant have numerous biological activities such as antitumor, antioxidative, and anti-inflammatory activities [9]. Ramsewak et al. (1999) had reported that carbazole alkaloids obtained from *M. koenigii* show cytotoxic capacity [11]. Even though many carbazole alkaloids have been isolated and identified from the different parts of *M. koenigii*, girinimbine was the first [12]. The effect of carbazole alkaloids in cancer has been studied in detail [13]. Recently, girinimbine and some structurally similar compounds isolated from the *M. koenigii* plant had showed that the anticancer effect of this compound involves apoptosis and free radical scavenging [14, 15]. But the involvement of proteins implicated in the intrinsic and/or extrinsic pathways including other apoptosis proteins has not been studied yet. In an effort to understand the mechanism behind the anticancer traditional pharmacological claim of this plant and to know the role of these proteins in cell death, we used A549 cells, which were shown to undergo apoptosis following exposure to girinimbine.

## 2. Materials and Methods

### 2.1. Isolation of Girinimbine

**Figure 1:**
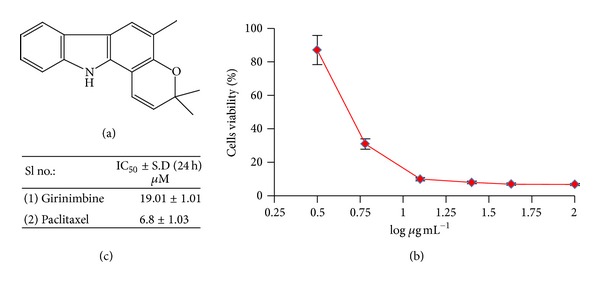
Effects of girinimbine on cell viability in A549 cells. (a) The chemical structures of girinimbine. (b) The cell viability of cells after 24 h of girinimbine treatment. Each point is the mean ± SD of three independent experiments. (c) IC_50_ values of both girinimbine and paclitaxel (positive control).

The root of *Murraya koenigii*  (L.) Spreng was collected from Sik, Kedah, Malaysia, in June 2006. The experimental work on plant to obtain pure girinimbine (Figure 1) and its spectroscopic data has been reported previously [16]. Briefly, the air-dried sample (525 g) was ground and further extracted with solvents using microwave-assisted extraction (45°C, 300 W for one hour). This process gave a hexane extract (22.7 g), chloroform extract (11.7 g), and methanol extract (40.7 g). Part of the hexane extract (20.7 g) was subjected to column chromatography fractionation with a combination solvent system of hexane, ethyl acetate, and methanol in increasing polarity to give 40 fractions. Fractions 9–14 which were eluted from hexane : ethyl acetate (95 : 5 ≥ 92 : 8) were combined and rechromatographed over silica gel to yield a yellow solid which was then recrystallized with hexane to obtain white crystals of girinimbine (0.77 g). The melting point of girinimbine was 171–173°C [16]. The isolated compound (girinimbine) was characterized by infrared spectroscopy, mass spectrometry, and nuclear magnetic resonance (^1^H and ^13^C NMR). The confirmation of the compound as well as the purity was again checked using HPLC and LC-MS. Further information regarding the mass spectra and purity of girinimbine can be obtained from the supplementary information provided, available online at http://dx.doi.org/10.1155/2013/689865.

### 2.2. Cell Culture

A549, a human nonsmall cell lung cancer cell line, was purchased from ATCC (Rockville, MD, USA) and cultured in RPMI-1640 supplemented with 100 *μ*L/mL fetal bovine serum, 100 U/mL penicillin, and 100 mg/mL streptomycin sulfate. Cells were maintained at 37°C in a humidified atmosphere of 5% CO_2_. Cells were treated with the girinimbine dissolved in DMSO, while the untreated control cultures received only the vehicle (DMSO < 1%).

### 2.3. Cell Viability Assay

Cells were seeded in 96-well plates at a density of 1 × 10^4^ per well and treated with various concentrations of girinimbine for 24 h. Cells were then incubated with a medium containing 5 mg/mL 3-(4,5-dimethylthiazol-2-yl)-2,5-diphenyltetrazolium bromide (MTT) for another 4 h. The viable cell number was directly proportional to formazan production which then dissolved in DMSO and was measured by spectrophotometry at 563 nm in a microplate reader (Tecan Infinite M 200 PRO, Männedorf, Switzerland) [17].

### 2.4. Microscope Examination on Cellular Morphology

Apoptosis was monitored by annexin V labeling and fluorescence microscopy [18]. Treated and untreated cells were washed with PBS and then exposed with annexin V-fluorescein (BD Pharmingen, USA) for 15 mins. After a 488 nm excitation, green fluorescence was visualized and recorded at 515 nm. Phase contrast microscopic images from the same preparations were also obtained for symptoms of apoptosis such as cell shrinkage, ruffling, and blebbing of cell membrane as well as fragmentation of cells into small apoptotic bodies using a fluorescent microscope (Nikon TE 2000U fluorescence inverted microscope, Tallahassee, Florida).

### 2.5. Determination of Lysosomal Membrane Stability

Cells were assessed for lysosomal stability using acridine orange (AO) uptake assay. Briefly, cells were incubated with 7 *μ*M AO (Sigma, USA) and 12 *μ*M Hoechst 33342 (Invitrogen, Carlsbad, CA, USA), diluted in culture media for 15 mins at 37°C, and immediately observed and analyzed using the ArrayScan HCS system (Cellomics, PA, USA). Acridine orange is a metachromatic fluorochrome and a weak base that exhibits red fluorescence when highly concentrated in acidic lysosomes. 

### 2.6. Measurement of Reactive Oxygen Species Generation

The production of intracellular reactive oxygen species (ROS) was measured using 2′,7′-dichlorofluorescein diacetate (DCFH-DA) [19]. DCFH-DA passively enters the cell where it reacts with ROS to form the highly fluorescent compound dichlorofluorescein (DCF). Briefly, 10 mM DCFH-DA stock solution (in methanol) was diluted 500-fold in HBSS without serum or other additives to yield a 20 *μ*M working solution. After 24 h of exposure to girinimbine, the cells in the 96-well black plate were washed twice with HBSS and then incubated in 100 *μ*L working solution of DCFH-DA at 37°C for 30 mins. Fluorescence was then determined at 485 nm excitation and 520 nm emission using a fluorescence microplate reader (Tecan Infinite M 200 PRO, Männedorf, Switzerland).

### 2.7. Multiple Cytotoxicity Assay

Cellomics Multiparameter Cytotoxicity 3 Kit was used as described in detail previously [20]. This kit enables simultaneous measurements in the same cell of six independent parameters that monitor cell health, including cell loss, nuclear size and morphological changes, mitochondrial membrane potential changes, cytochrome c release, and changes in cell permeability. Plates were analyzed using the ArrayScan HCS system (Cellomics, PA, USA).

### 2.8. Image Acquisition and Cytometric Analysis

Plates with stained cells were analyzed using the ArrayScan HCS system (Cellomics, PA, USA). This system is a computerized automated fluorescence imaging microscope that automatically identifies stained cells and reports the intensity and distribution of fluorescence in individual cells. The Array-Scan HCS system scans multiple fields in individual wells to acquire and analyze images of single cells according to defined algorithms. In each well, 1,000 cells were analyzed. Automatic focusing was performed in the nuclear channel to ensure focusing regardless of staining intensities in the other channels. Images were acquired for each fluorescence channel, using suitable filters. Images and data regarding intensity and texture of the fluorescence within each cell, as well as the average fluorescence of the cell population within the well, were stored in a Microsoft SQL database for easy retrieval. Data were captured, extracted, and analyzed with ArrayScan II Data Acquisition and Data Viewer version 3.0 (Cellomics).

### 2.9. Human Apoptosis Proteome Profiler Array

To investigate the pathways by which girinimbine induces apoptosis, we performed a determination of apoptosis-related proteins using the Proteome Profiler Array (RayBio Human Apoptosis Antibody Array Kit, RayBiotech, USA), according to manufacturer's instructions. In short, the cells where treated with 19 *μ*M girinimbine. 300 *μ*g proteins from each sample and were incubated with the human apoptosis array overnight. The apoptosis array data were quantified by scanning the membrane on a Biospectrum AC ChemiHR 40 (UVP, Upland, CA) and analysis of the array image file was performed using image analysis software according to the manufacturer's instruction.

### 2.10. Measurement of Caspases 8, 9, and 3/7 Activities

Caspases 3/7, 8, and 9 activity was measured using luminescence-based assay, Caspase-Glo 8 Assay, Caspase-Glo 9 Assay, and Caspase-Glo 3/7 Assay (Promega). 1 × 10^4^ cells were cultured in 96-well culture plates in 50 *μ*L of RPMI 1640 supplemented with 10% FBS and incubated for 24 h. Cells then were treated with different concentrations of girinimbine and incubated for 24 h. At the end of incubation, 100 *μ*L of assay reagent was added to be incubated for 1 h at room temperature. Luminescence was measured using a microplate reader (Tecan Infinite M 200 PRO, Männedorf, Switzerland).

### 2.11. Statistical Analysis

From several independent measurements means and standard deviations were calculated. Testing for significant differences between means were carried out using the one-way ANOVA and Dunnett's post-test at probabilities of error of 5% and 1%.

## 3. Results

### 3.1. Antipoliferative Activity

The sigmoidal dose response curves of girinimbine in the end-point assays are shown in Figure 1. Cell viability was analyzed using the MTT assay, which measures the metabolic activity of cell. In the A549 cells treated with girinimbine, metabolic activity decreased followed by 24 h treatment; meanwhile in the control plate, cell viability and metabolism were not affected. Figure 1(c) summarizes the IC_50_ values from MTT. Meanwhile, even at 380 *μ*M girinimbine could not exhibit any sign of toxicity in WRL-68, a normal (data not shown) which was employed in this study to investigate the specificity of cytotoxicity.

### 3.2. Apoptotic Mode of Cell Death

**Figure 2:**
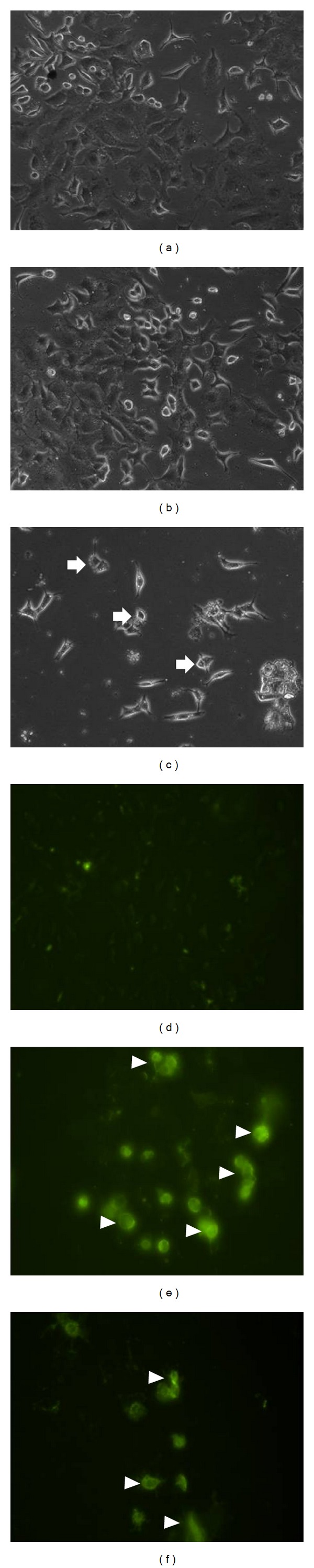
Effects of girinimbine on the morphological changes of A549 cells after 24 h treatment. Apoptosis was monitored by phase contrast ((a) control, (b) treatment 9 *μ*M, and (c) treatment 19 *μ*M) and fluorescence microscopy ((d) control, (e) treatment 9 *μ*M, and (f) treatment 19 *μ*M). Arrows indicate the cell shrinkage, ruffling, and blebbing of cell membrane while arrowheads indicate the annexin V binding to phosphatidylserine. Magnification 20X.

Data obtained from fluorescence detection of A549 cells together with the phase contrast microscopic images are shown (Figure 2). Apoptosis was clearly detected by the phosphatidylserine externalization on the treated cells. Phase contrast microscopic pictures showed a clear morphological change of treated cells which were observed after 24 h of treatment with girinimbine. A549 cells were seen to have shrunk in size and there was ruffling and blebbing of cell membranes, thus suggesting that the cells were undergoing apoptosis (Figure 2(c)). 

### 3.3. Girinimbine Initiates Lysosomal Membrane Permeabilization

**Figure 3:**
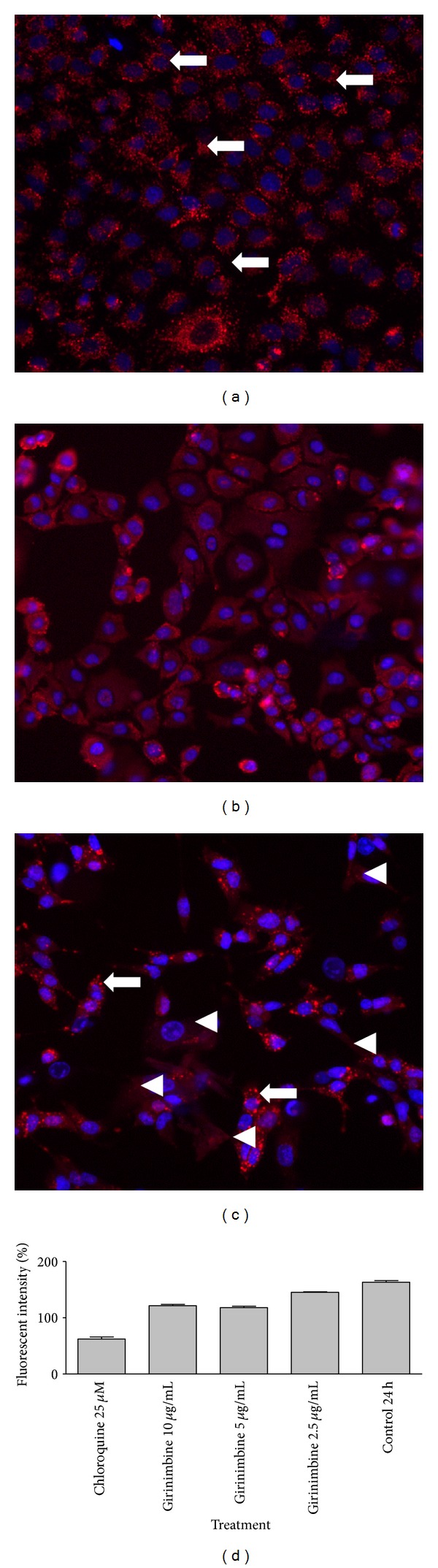
Effects of girinimbine on A549 cells lysosome acidic environment. Cells were incubated with different concentrations of girinimbine for 24 h, stained with acridine orange and analyzed by ArrayScan HCS system. Control group corresponds to untreated cells. Acridine orange is a metachromatic fluorochrome and a weak base that exhibits red fluorescence when highly concentrated in acidic lysosomes. (a) Normal lysosome acidic environment (arrow). Notice the weak lysosome staining pattern and cytoplasm acidification (arrowhead) in cells treated with chloroquine (b) and girinimbine (c), indicating lysosomal membrane permeabilization. Magnification 20X. The mean fluorescent intensity produced by the acridine orange was quantitatively measured (Figure 3(d)).

Recent studies have shown that lysosomal membrane permeabilization (LMP) is an early and perhaps initiating event in apoptosis triggered by ligation of death receptors, lysosomotropic agents, oxidative stresses, or serum withdrawal [21, 22]. To evaluate the lysosome acidification pattern, the cells were analyzed by acridine orange staining, a known pH indicator. While untreated A549 cells displayed strong granular acridine orange staining, cells treated with girinimbine presented weak lysosome staining pattern and cytoplasm acidification, indicating lysosomal membrane permeabilization (Figures 3(a)–3(c)). Girinimbine induced dose-dependent decrease of fluorescence intensity of lysosomal staining, which was well compared with chloroquine, a known inhibitor of lysosome function, was included as a positive control (Figure 3(d)).

### 3.4. ROS Assay

**Figure 4:**
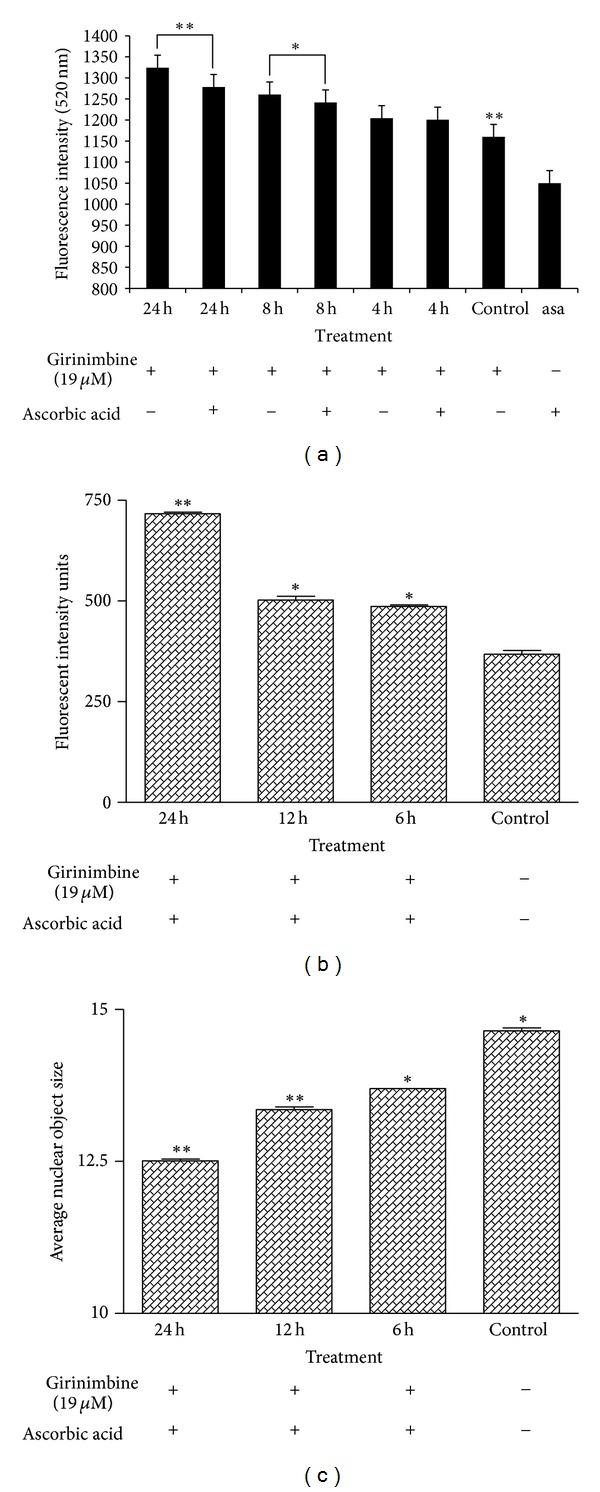
Effects of girinimbine on A549 cells ROS generation. (a) DCF-fluorescence intensity after ascorbic acid and 19 *μ*M of girinimbine exposure at 4, 8, and 24 h. Rate of apoptosis in terms of fluorescent intensity (b) and average nuclear object size (c) of nucleus of the girinimbine treatment was pretreated with 100 mM ascorbic acid. Values are mean ± SD from three independent experiments. Triplicates of each treatment group were used in each independent experiment. The statistical significance is expressed as **P < 0.01; *P < 0.05. Fluorescent intensity units directly represent the amount of ROS production.

Numerous investigations have documented that oxidative stress-mediated cellular changes are frequently induced in cells exposed to cytotoxic drugs, UV, or gamma irradiation [23, 24]. We examined whether girinimbine affects the cellular levels of peroxide by measuring the changes in the fluorescence using DCF-DA. As shown in Figure 4(a), treatment with girinimbine markedly increased the DCF-DA-derived fluorescence (520 nm). This girinimbine-mediated increase in fluorescence was markedly inhibited by pretreatment with antioxidant ascorbic acid. Then, we next asked whether ROS generation induced by girinimbine is directly associated with the induction of apoptosis. Chromatin condensation was measured in the cells which were pretreated with ascorbic acid, using 12 *μ*M Hoechst 33342. The results shown in Figure 4(b) clearly exhibited that pretreatment with ascorbic acid could not prevent the apoptosis at various time periods. Moreover, the data clearly shows the rapid decrease in the nuclear area and the increase in fragmentation, upon treatment with 19 *μ*M girinimbine (Figure 4(c)). 

### 3.5. Multiparameter Cytotoxicity Analysis

**Figure 5:**
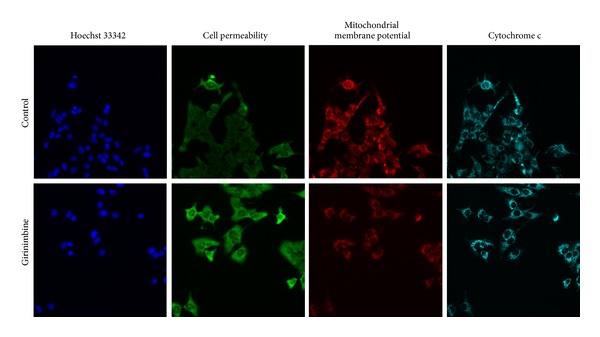
Representative images of A549 cells treated with medium alone and 19 *μ*M of girinimbine and stained with Hoechst for nuclear, cell permeability dye, mitochondrial membrane potential dye, and cytochrome c. The images from each row are obtained from the same field of the same treatment sample. A549 produced a marked reduction in mitochondrial membrane potential and marked increases in membrane permeability and cytochrome c. Magnification 20X.

**Figure 6:**
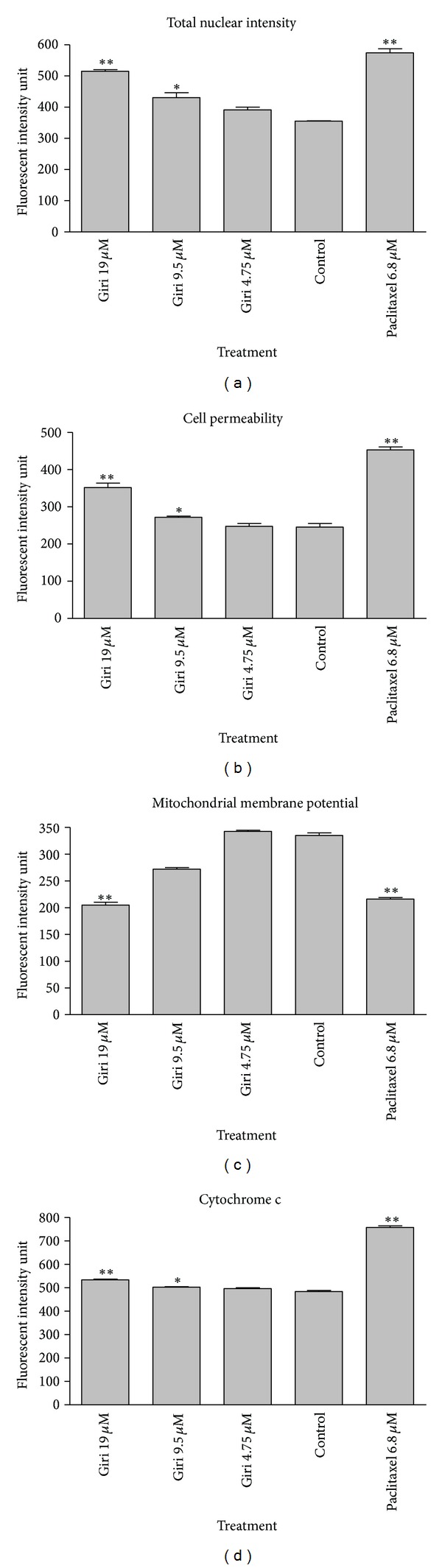
Quantitative analysis of girinimbine mediated apoptosis parameter. Changes in total nuclear intensity (a), cell permeability (b), mitochondrial membrane potential (c), and cytochrome c localization (d) were measured simultaneously in A549 cells. Following treatment with girinimbine, we saw statistically significant increase in total nuclear intensity, increased cell permeability, loss of mitochondrial membrane potential, and cytochrome c release from mitochondria. Each experiment was performed at least two times. Results are expressed as the means ± SD. Statistical analysis was performed with one-way analysis of variance (ANOVA) using GraphPad Prism software (version 4.0; GraphPad Software Inc., San Diego, CA). Statistical significance is expressed as **P < 0.01; *P < 0.05.

This assay enables simultaneous measurement of several cell-health parameters: nuclear morphology, DNA content, cell membrane permeability, and cytochrome c localization and release from mitochondria. Typical cytotoxic changes are illustrated in Figures 5 and 6. Girinimbine induced decreases in cell number; nuclear area intensity and plasma membrane permeability were significantly higher in the treated cells (*P* < 0.01) and mitochondrial membrane potential reduction was observed significantly at 19 *μ*M (*P* < 0.01). The release of cytochrome c was observed significantly in 9 *μ*M and 19 *μ*M with *P* < 0.05 and *P* < 0.01, respectively. These effects occurred more rapidly and followed a dose-response pattern. Cytotoxic effects were considered to occur only when the rate of change of fluorescence was distinctly greater than for the negative controls.

### 3.6. Effect of Girinimbine on Apoptotic Markers

**Figure 7:**
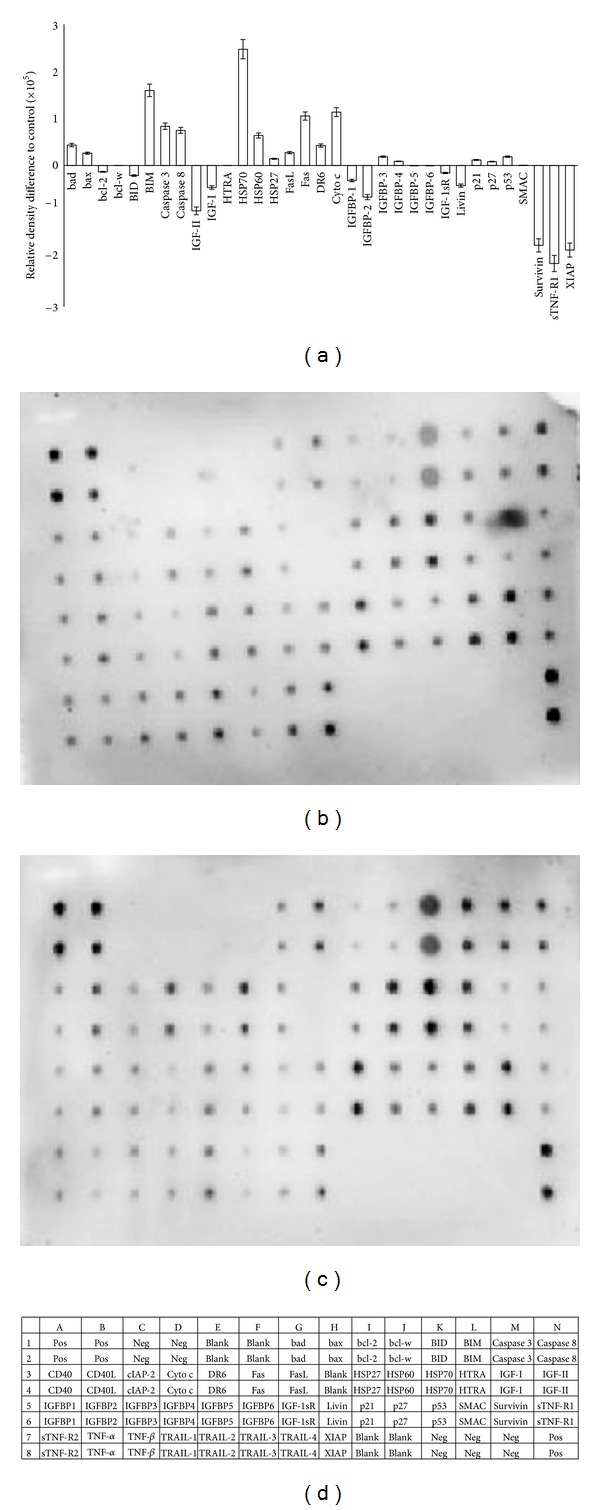
Cells were lysed and protein arrays were performed. Cells was treated with 19 *μ*M girinimbine for 24 h and the whole cell protein was extracted. Equal amount of (300 *μ*g) of protein from each sample was used for the assay. Quantitative analysis in the arrays showed differences in the apoptotic markers (a). Representative images of the apoptotic protein array are shown for the control (b), treatment (c), and the exact protein name of each dot in the array (d).

After girinimbine (19 *μ*M) exposure, A549 cells were lysed and apoptotic markers where screened using a protein array. All major markers which are involved in both intrinsic and extrinsic pathways were induced on treatment. As shown in Figure 7, girinimbine treatment significantly increased the expression of caspase 8, suggesting the activation of the death receptor pathway. In addition, the major protein involved in the extrinsic pathway such as Fas and FasL also regulates the treatment. Moreover the involvement of mitochondria in the cell death was evident by the regulation of the Bcl-2 family of proteins such as Bad, Bax, Bcl-2, and Bim. Besides, the Bcl-2 family member Bid was found to be cleaved as well, suggesting a potential cross-talk between the death receptor and the mitochondrial pathway. The treatment also resulted in a reduction in the level of expression of the inhibitor of apoptosis XIAP as well as survivin. P53 as well as the cell proliferation repressor proteins, p27 and p21, and the heat shock proteins such as antiapoptotic HSP60, HSP70, and HSP27, which are a result of oxidative stress in the cell, were also induced. Insulin/IGF-1 signaling related protein expression has been observed on treatment. Downregulation of antiapoptosis (IGF-I, IGF-II, IGFBP1, IGFBP2) and upregulation of proapoptosis proteins (IGFBP3, IGFBP4) were observed. 

### 3.7. Caspase Depended Apoptosis in Girinimbine Treated A549 Cells

**Figure 8:**
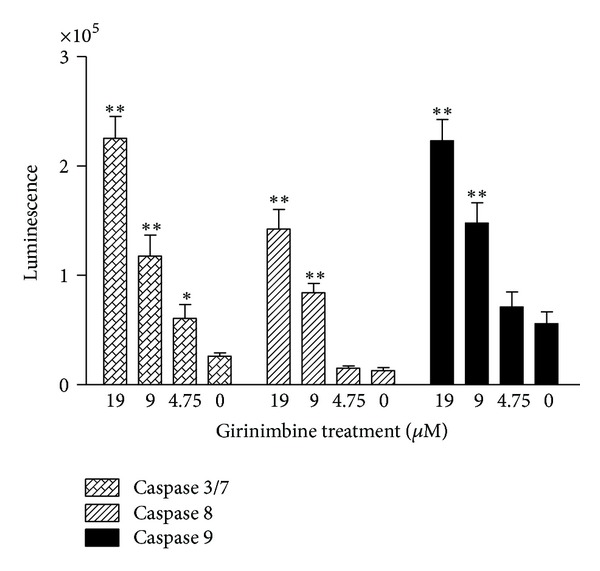
Effect of girinimbine on activation of caspases 3/7, 8, and 9. Cells were treated with girinimbine at the indicated concentrations for 24 h and activity was determined by using a luminescent enzyme assay kit. Each point represents the mean ± SD of three different experiments. Statistical analysis was performed with one-way analysis of variance (ANOVA) using GraphPad Prism software (version 4.0; GraphPad Software Inc., San Diego, CA). Statistical significance is expressed as **P < 0.01; *P < 0.05.

The involvement of the caspase cascade in the girinimbine mediated cell death was confirmed. The treatment with 19 *μ*M was significant at *P* < 0.01 for caspase 3/7 and 9 with 8- and 5-fold increase, respectively. Meanwhile the caspase 8 showed 10-fold increase at the maximum treatment concentration (19 *μ*M, *P* < 0.01) (Figure 8). These results are in parallel with the protein array and indicate that the signaling cascade leading to apoptosis in girinimbine-treated cells involves both intrinsic and extrinsic pathways.

## 4. Discussion

Apoptosis is a normal physiological process that plays a vital role in numerous normal functions [4]. Furthermore, it is an active physiological process causing cellular self-destruction that comprises specific morphological and biochemical changes in the nucleus and cytoplasm [25]. The involvement of an energy-dependent cascade of molecular events makes the mechanism of apoptosis very highly complex and sophisticated [26]. Apoptosis is regulated by two major pathways: the extrinsic and the intrinsic pathways [27]. Nevertheless, there is evidence suggesting that both the pathways are linked and that molecules involved in the pathways can influence one another [28].

The current study found that girinimbine, a carbazole alkaloid from the roots of *M. koenigii*, can inhibit cell proliferation selectively in a dose dependent manner in A549 cells. The morphological observation was conducted to explore whether the cytotoxic effect was related with the apoptotic process, and it was found that the cell death induced by girinimbine exhibited a clear morphological sign of apoptosis, as this is an important property of a candidate anticancer drug [29]. Followed by 24 h treatment, cell shrinkage, ruffling, and blebbing of cell membranes were observed. These morphological features were more evident while we analysed one of the key features of apoptosis, which is the change in plasma membrane structure by surface exposure of phosphatidylserine (PS), while the membrane integrity remains unchallenged [30]. The incorporation of annexin V in to the same treated cells showed distinctive form of binding between the externalized PS and Annexin V, while cells from test flasks showed more evidence of apoptosis than those grown under control conditions (Figure 2).

Translocation of lysosomal hydrolases from the lysosomal lumen to the rest of the cell followed by the impairment in the lysosomal membrane function is known as lysosomal membrane permeabilization (LMP) [31]. Often, these hydrolases facilitate apoptosis by inducing mitochondrial outer-membrane permeabilization and caspase activation [32]. In our results, the main LMP consequence such as weak lysosome acidification pattern and cytoplasmic acidification was clearly evident (Figure 3). Being a weak base chloroquine, acts as lysosomotropic drug that raises intralysosomal pH. Hence we have monitored the release of liposomal content by using chloroquine as a positive control. Moreover, in the Figure 3, it is very clear that cells treated with girinimbine resented a weak lysosome acidification pattern followed by an increase in cytoplasmic acidification. Bearing in mind the significance of acidic lysosome environment for proteins and organelles degradation, it can be pointed out that; autophagy may not the main event of cell death. 

Destabilization of the lysosomal membrane may be caused by increased production of ROS via massive peroxidation of membrane lipids [33]. Moreover, many studies had showed the relation between ROS and apoptosis induced in cells exposed to cytotoxic drugs [34]. Mitochondrial ROS are used as active mediators in the regulation of cell death [35]. In our study, the generation of ROS led to the upregulation of cytoprotective protein markers HSP 60, 70, and 27.  Figure 4 clearly shows that girinimbine exposure leads to increase of ROS accumulation, but inhibition of ROS by ascorbic acid did not prevent girinimbine-induced apoptosis, indicating that ROS generation is not critical for the induction of apoptosis by girinimbine in A549 cells. Moreover, the treatment with girinimbine significantly decreases the cell number, nuclear area, and cell membrane permeability as shown by the multiparameter apoptosis analysis. In addition, the complex role of mitochondria in A549 cell apoptosis was investigated. Mitochondria have been described to play a central role in the apoptotic process due to the mitochondrial proteins ability to activate cellular apoptotic programs directly [36]. Detection of changes in mitochondrial membrane potential (Ψm) was carried out as it is assumed that its disruption is the onset of mitochondrial membrane transition pores (MPTP) formation. The present study revealed that girinimbine may act on mitochondria, causing loss of Ψm and subsequent apoptosis. This kind of Ψm reduction and generation of MPTP must facilitate the relocalization of apoptogenic proteins from one subcellular compartment such as mitochondria to cytoplasm to gain access to their substrates or interacting partners [37].

The multi cytotoxicity assay shows that girinimbine induced cell death could be through the classical mitochondrial pathway with cytochrome c release and caspase-dependent apoptosis. The translocation of cytochrome c, a component of the mitochondrial electron transfer chain, was significantly increased upon girinimbine treatment. In the mitochondria mediated apoptosis, cytochrome c in turn binds to Apaf-1 to form a complex, which triggers its oligomerization to form the apoptosome. The caspase-9 holoenzyme is then forms due to the binding of procaspase-9 to the apoptosome and sequentially cleaves and activates the downstream caspases, such as caspase-3 [38]. Our findings on the upregulation of caspase 3/7, caspase 9, reduction in Ψm, and release of cytochrome c to the cytosol substantiate this theory and strongly support the involvement of mitochondria in the apoptosis induced by girinimbine in A549 cells. Suppression of apoptosis may promote the cancer development by inhibiting the chemotherapy as well as other forms of inducers of cell death [39]. Regulation of apoptosis has been censoriously dependent on several genes, which have been identified earlier and includes XIAP-a member of the IAP family. It has the capacity to inhibit the activation of caspases 3, 7, and 9 [40]. In our study, we found that girinimbine treatment of A549 cells caused inhibition of XIAP, but SMAC, an antagonist to XIAP, could be found to be slightly regulated in this treatment concentration. This is considered as a key event in the execution of cell death, which is regulated mainly by proteins of the Bcl-2 family [41, 42].

Critical issues in apoptosis include the dominance of anti-versus proapoptotic Bcl-2 members [43]. Girinimbine is found to induce apoptosis in the A549 cells, involving downregulated Bcl-2 expression. This downregulation was concomitant with the upregulation of Bad, Bax, and Bim, which inhibits antiapoptotic proteins and render the cells more susceptible to apoptogenic stimuli (Figure 7). The release of cytochrome c found in our study could be due to the up regulated Bax, since Bax in association with adenine nucleotide translocator within the permeability transition pore complex will increase the mitochondrial membrane permeability, and thereby discharge a number of apoptogenic molecules into the cytosol [44]. In addition to this, upregulation of the tumor suppressor protein p53, which has a critical role in regulation of the Bcl-2, and p27, and p21, draws attention to the involvement of cell cycle arrest in the apoptosis process. Alternatively we found the involvement of extrinsic pathway proteins such as Fas and FasL upregulation, and related upregulation of caspase 8 (Figure 7). Moreover, the luminescent based caspase 8 assay had showed 10 fold differences than the control. Apart from this, we found that there is a downregulation of Bid happening too. Since mitochondrial damage in the Fas pathway of apoptosis is mediated by the caspase 8 cleavage of Bid, [45] the findings here shows the cross talk between intrinsic and extrinsic pathways.

 The protein array results exhibited a significant reduction of both IGF-I and IGF-II. IGF-I has been shown to exert strong mitogenic and antiapoptotic effects in a variety of normal and cancerous cells, including lung cancer cell lines [46]. Moreover the over expression of IGFBP3 is closely associated with the downregulation of IGF-I and its mitogenic activity [47]. It is known that IGFBP-2 and IGFBP-1 are involved in the regulation of cell migration, apoptosis, and cell growth [48]. Both are reported to be markedly overexpressed in many tumors and tumor cell lines. And it is assumed that elevation of the IGFBP-2 production is part of a mechanism to compensate for the mitogenic and antiapoptotic effects of tumor-derived IGFs [49]. Our data clearly showed the downregulation of IGFBP1 and IGFBP2 concomitant with upregulation of IGBP3.

Our results provided a new insight into the mechanism of chemotherapeutic properties of *Murraya koenigii. *In addition, these study findings demonstrate that girinimbine mediates its apoptotic effects through both intrinsic and extrinsic pathway, which is depended on caspase mediation. Moreover, the upregulation of p53 as well as the cell proliferation repressor proteins, p27 and p21, and the significant role of insulin/IGF-1 signaling were also identified. These results indicate that girinimbine could be a candidate for a novel anticancer agent. Therefore, more in-depth *in vitro* and *in vivo* studies are currently going on in our laboratory.

## Supplementary Material

The obtained girinimbine was checked for its purity by using HPLC. The obtained single peak of girinimbine confirmed that the compound is more than 98.5 % pure.
