# Supplementary figures and images for: Apoptosis Effect of Girinimbine Isolated from Murraya koenigii on Lung Cancer Cells In Vitro
Source: Evid Based Complement Alternat Med. 2013 Mar 13;2013:689865. doi: 10.1155/2013/689865 (PMC3610346; doi:10.1155/2013/689865)

PDA Ch1 220nm 4nm  
mAU

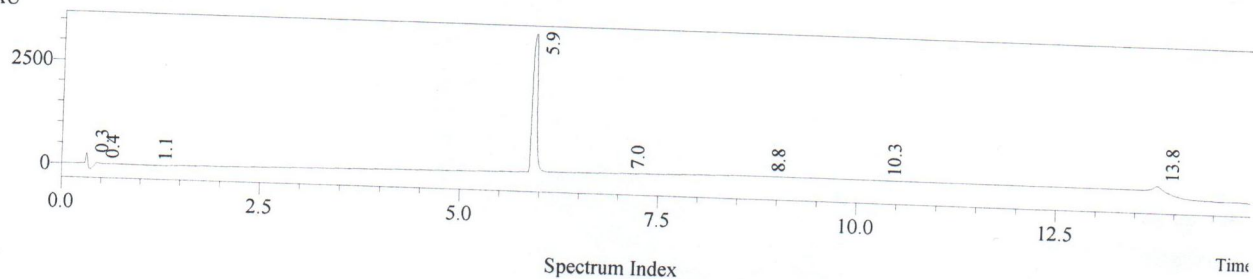

PDA Ch2 254nm 4nm  
mAU

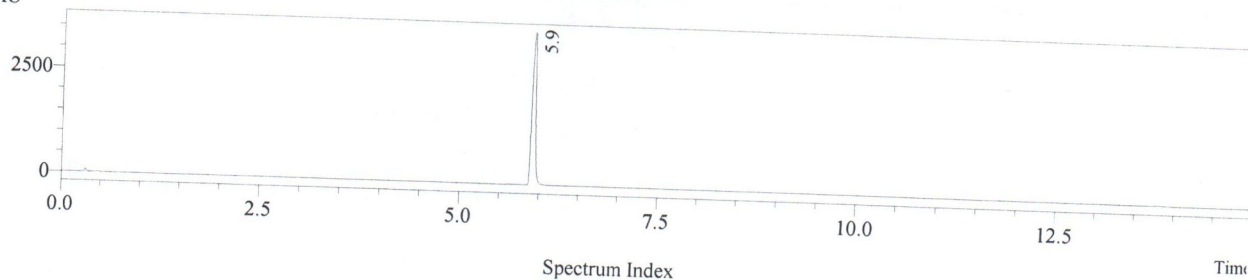

PDA Ch3 350nm 4nm  
mAU

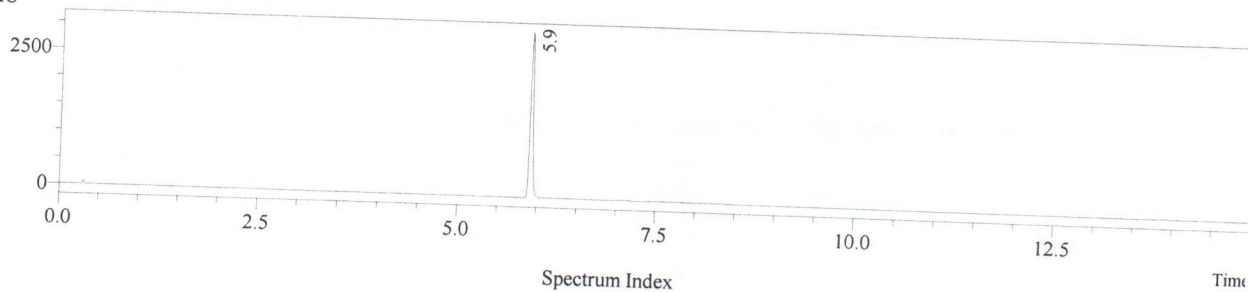

PDA Ch4 450nm 4nm  
mAU

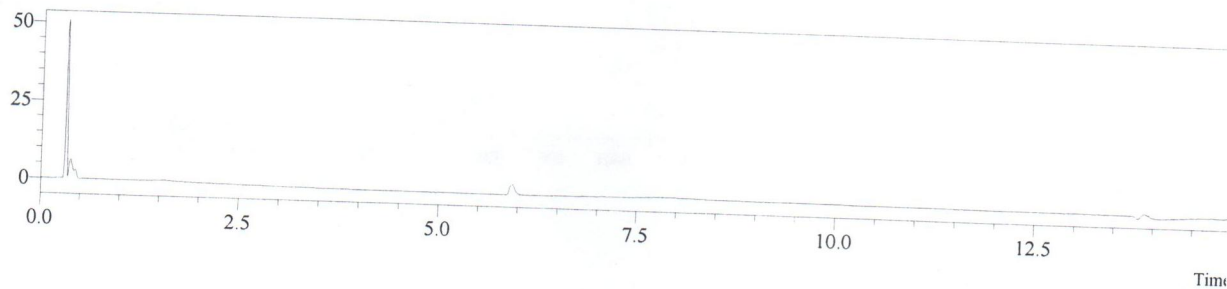

Supplement: Supplementary file 1 — The obtained girinimbine was checked for its purity by using HPLC. The obtained single peak of girinimbine confirmed that the compound is more than 98.5 % pure. [file 689865.f1.pdf]
